# Neurophysiological responses to unpleasant stimuli (acute electrical stimulations and emotional pictures) are increased in patients with schizophrenia

**DOI:** 10.1038/srep22542

**Published:** 2016-03-03

**Authors:** Céline Z. Duval, Yannick Goumon, Véronique Kemmel, Jürgen Kornmeier, André Dufour, Olivier Andlauer, Pierre Vidailhet, Pierrick Poisbeau, Eric Salvat, André Muller, Ayikoé G. Mensah-Nyagan, Catherine Schmidt-Mutter, Anne Giersch

**Affiliations:** 1 INSERM U-1114, Fédération de Médecine Translationnelle de Strasbourg (FMTS), Département de Psychiatrie, Hôpitaux Universitaires de Strasbourg; 1, place de l’Hôpital, 67000 Strasbourg, France; 2 Fondation FondaMental, Créteil, France; 3 Fondation APICIL, 21, place Bellecour, 69002 Lyon, France; 4 Institut des Neurosciences Cellulaires et Intégratives, CNRS UPR 3212, 5, rue Blaise Pascal, 67084 Strasbourg, France; 5 INSERM U-1119 Biopathologie de la Myéline, Neuroprotection et Stratégies Thérapeutiques, Université de Strasbourg, Bâtiment 3 de la Faculté de Médecine, 11 rue Humann, 67000 Strasbourg, France; 6 Laboratoire de Biochimie et Biologie Moléculaire, Hôpitaux Universitaire de Strasbourg, 67098 Strasbourg, France; 7 Institute for Frontier Areas of Psychology and Mental Health, 79098 Freiburg, Germany; 8 University Eye-Hospital, Killianstraße 5, 79106 Freiburg, Germany; 9 Laboratoire de Neurosciences Cognitives et Adaptatives, UMR 7364, Université de Strasbourg, CNRS, Strasbourg, France; 10 EA 481 Laboratoire de Neurosciences, Université de Franche-Comte, 1 place du maréchal Leclerc, 25030 Besançon Cedex, France; 11 Centre ‘Evaluation et de Traitement de la Douleur (CETD) du CHRU, Hôpital de Hautepierre, 1 av Moliere, 67078 Strasbourg, France; 12 INSERM Centre d’investigation clinique-1434, CHRU, 1, place de l’Hôpital, 67000 Strasbourg, France

## Abstract

Patients with schizophrenia have often been described as insensitive to nociceptive signals, but objective evidence is sparse. We address this question by combining subjective behavioral and objective neurochemical and neurophysiological measures. The present study involved 21 stabilized and mildly symptomatic patients with schizophrenia and 21 control subjects. We applied electrical stimulations below the pain threshold and assessed sensations of pain and unpleasantness with rating scales, and Somatosensory Evoked Potentials (SEPs/EEG). We also measured attention, two neurochemical stress indices (ACTH/cortisol), and subjective VEPs/EEG responses to visual emotional stimuli. Our results revealed that, subjectively, patients’ evaluations do not differ from controls. However, the amplitude of EEG evoked potentials was greater in patients than controls as early as 50 ms after electrical stimulations and beyond one second after visual processing of emotional pictures. Such responses could not be linked to the stress induced by the stimulations, since stress hormone levels were stable. Nor was there a difference between patients and controls in respect of attention performance and tactile sensitivity. Taken together, all indices measured in patients in our study were either heightened or equivalent relative to healthy volunteers.

It is not always clear what patients with schizophrenia can or cannot feel. It is generally acknowledged that patients are very sensitive to stressful events, which is a possible vulnerability factor^1^. However, co-existing with this observation are descriptions of reduced sensitivity to the outside world^2^,^3^. The contradictory observations make it difficult to discern how patients react to different stimuli and situations, especially in the case of unpleasant information.

The hypothesis that patients are insensitive to information about the world outside is based on clinical observations, according to which they are less reactive to pain and negative pictures. The most spectacular observations are the severe self-mutilations observed in acute phases of schizophrenia^4^. Other observations concern chronic patients, who fail to signal pain in the case of somatic diseases^5^,^6^. Additionally, population-based studies have shown that chronic pain and schizophrenia comorbidity is rare^7^,^8^,^9^.

However, although experimental studies and meta-analyses generally suggest reduced rather than heightened pain sensitivity (reviewed in^10^,^11^,^12^,^13^,^14^,^15^,^16^), some studies suggest a more complex response pattern in patients. For instance, it has been suggested there is a dissociation between responses to acute and prolonged pain in schizophrenia, with more sensitivity to acute pain, and less sensitivity to prolonged, repetitive pain^17^. The explanation for this contrast might be a phenomenon of sensitization in the case of repeated and high frequency pain stimulation, resulting in increased pain sensitivity in healthy subjects^18^. Sensitization has been shown to be impaired in patients with schizophrenia^14^,^17^,^19^, which might contribute to reduced pain sensitivity compared with matched controls. A second source of difficulty has to do with the fact that, overall, the majority of experimental studies on pain in schizophrenia rely on subjective responses. More objective physiological evidence is so far rare. This is all the more important insofar as a dissociation between subjective and objective measures has often been questioned^11^,^13^, with patients being impaired for subjective but not objective measures. Here we used EEG, the most time-accurate and sensitive measure of subjects’ reactions to pain.

To the best of our knowledge, only one study has ever explored SEP (somatosensory evoked potentials) responses to pain in patients with schizophrenia^20^. The authors described reduced subjective sensitivity to electrical stimulation, as well as lower amplitudes of the evoked potentials 100 ms after stimulation. However, the interval between stimulations was only 1 second, and, as described above, short intervals can result in a sensitization phenomenon. Hence, it is still unclear whether in patients the response to unpleasant and painful stimulation is always decreased, or whether chronic patients can be sensitive to such stimulation, at least when it is acute. The latter would be evidence of a form of persisting vulnerability in chronic patients, and would also have everyday implications, inasmuch as it would mean patients are not always as insensitive as they would appear to be. In the present study, we measured EEG responses to acute pain. For ethical reasons, and since we expected enhanced responses, we used mild electrical stimulations, i.e. below the pain threshold.

In addition we explored responses to emotional pictures, which represent a different type of unpleasant stimuli. Emotion is a component of pain, and its evaluation is necessary for interpreting the results on pain. While patients with schizophrenia are known to be poor at expressing emotions and interacting with other people^21^, recent studies suggest they experience emotions like controls^21^,^22^, but have difficulty regulating them^23^. Pain may not be controlled in the same way as emotions, but its processing also includes complex gating mechanisms^24^,^25^, which might be affected in patients with schizophrenia. Our main objective was thus to check whether the patients’ physiological and subjective responses to unpleasant electrical stimulations and pictures would be increased or decreased. We also checked for a possible involvement of stimulus-induced stress. Patients’ hypersensitivity to stress has been associated with excessive activity of the hypothalamic-pituitary-adrenal axes (review in^21^). This hypersensitivity may mediate an excessive response to unpleasant stimulations, a possibility we explored by measuring activity in the corticotropic axes. Finally, we checked for a possible impact of cognitive ability by evaluating attention with a continuous performance task.

## Results

### Pain perception

#### Subjective evaluation

As expected, the electrical stimulations were rated as significantly more painful and unpleasant in the high intensity condition (1800 μA) than in the low intensity condition (1300 μA) (24.6 vs. 17.9 for pain, F[1,40] = 25, p < 0.001, Cohen’s d = 0.36; and 56 vs 49.2 for unpleasantness, F[1,40] = 25.9, p < 0.001, Cohen’s d = 0.42). Patients’ pain ratings were slightly, but not significantly, higher than those of controls (26.7 vs. 15.8, F[1,40] = 2.4). Unpleasantness ratings did not differ between patients and controls (54.6 vs. 57.5 F[1,40] = 0.07), and individual analyses show there is no sub-group of patients showing reduced sensitivity (see Fig. S1 in Supplementary Information).

### Electrical stimulations-related SEP recordings

#### N1/P1 and N2/P2

No significant differences were observed between the two groups for the components N1/P1 or N2/P2 after electrical stimulations (Table 1).

### P50/P1*

**Figure 1:**
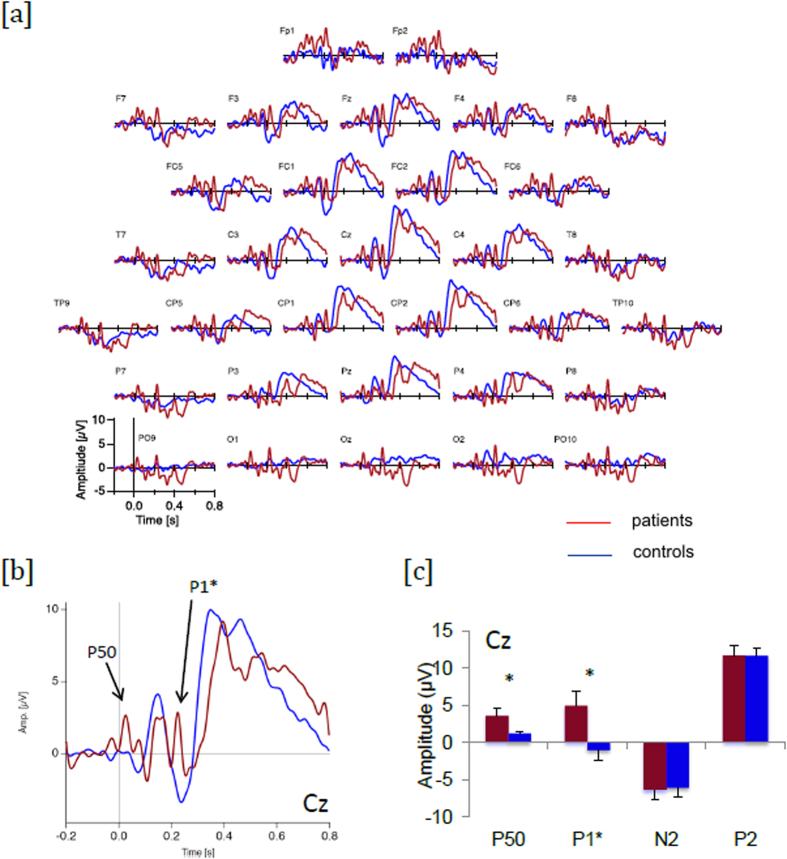
Representation of the pain-related SEP in patients with schizophrenia (red) and control subjects (blue) after mild painful electrical stimulations at 1800μV. (**a**) overall distribution of SEP traces on a schematic skull with 32 electrodes (**b**) SEPs at electrode Cz (**c**) amplitude of the 4 main peaks, in patients (red) and controls (blue).

We observed two early positive deflections which were present in patients with schizophrenia but absent in the control subjects. The earliest one looked like a P50, and a second one, which we shall call a P1*, was observed between 210 and 250 ms after stimulation onset. These deflections were strongest at fronto-central electrodes, and we focused on electrodes Cz and Fz for further statistical analysis of the two components (Fig. 1 and Table 1).

**Figure 2:**
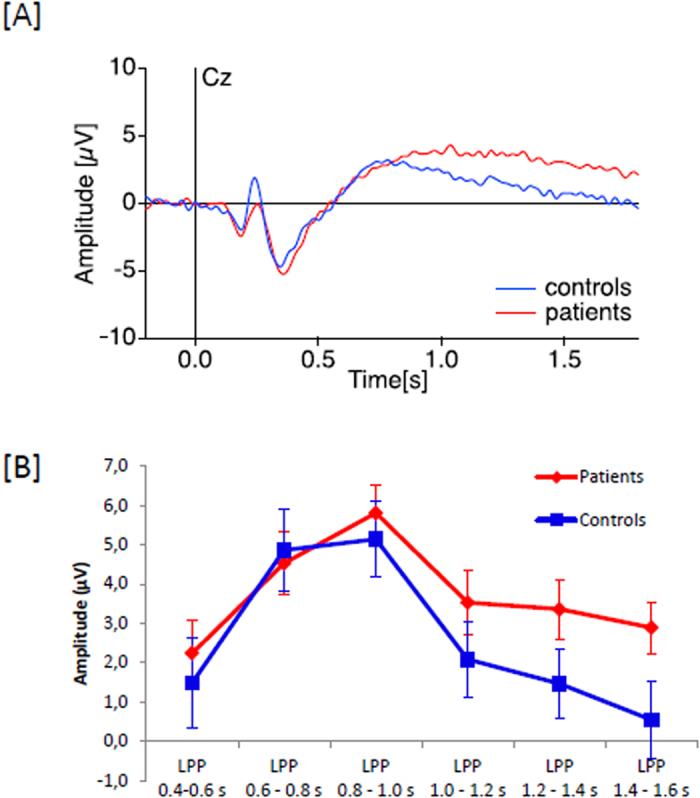
Representations of the visual ERP in patients with schizophrenia (red) and control subjects (blue) after visual stimulations with pictures with different emotional valences. (**A**) ERP traces of patients and controls at electrode Cz, averaged over all picture types. (**B**) Time course of the LPP amplitude in each group based on individual peak analysis.

The amplitude of both signals differed significantly across groups for 1800 μA stimulations. The amplitude of the P50 was significantly greater in patients than controls at the two sites Cz and Fz. This effect was not significant at 1300 μA (Fig. 2 and Table 2). By examining the results individually, we were able to rule out the possibility that the P50 is an artifact due to the electrical stimulations. The P50 started after the stimulation period and was present in the majority of patients (see Fig. S2 in Supplementary Information). As regards the P1*, its amplitude was significantly greater in patients than controls for both intensities at the Fz site. At the Cz site this difference was significant only for 1800 μA intensities (Fig. 1 and Table 1).

The figure may suggest a slight time lag in patients compared to controls. However, this difference was not significant (see Supplementary Information for details).

#### Tactile sensitivity

There was no significant group effect for tactile sensitivity [F(9,360) = 0.37, p = 0.95] (see Fig. S3 in Supplementary Information).

### Emotion perception

#### Subjective evaluation

We analyzed valence and arousal ratings of images as a function of the within-group factor ‘emotion’ (neutral, negative low arousal and negative high arousal). There was a main effect of ‘emotion’ for both valence (F[2,72] = 108.5, p < 0.001) and arousal (F[2,72] = 42.5, p < 0.001). No group effect was found for valence (F < 1), but overall patients rated the three different kinds of pictures as significantly more arousing than healthy control subjects (4.4 vs. 3.3, F[1,36] = 5.04, p < 0.05, Cohen’s d = 0.57).

#### Visual evoked potentials

Like for subjective responses, there was no interaction between emotion and group for evoked potentials (F < 1). We thus averaged the results over the three different picture types.

The results showed an interaction between group and LPP intervals over time [F(5,180) = 2.44, p < 0.05)]. The amplitude of the LPP was greater in patients than controls at a late stage of the LPP starting at around 1200 ms in fronto-central regions (Fig. 2A,B and Table 2).

### ACTH and cortisol levels

Patients’ ACTH levels tended to be higher than controls’ [F(1,40) = 3.63, p = 0.06], but there was no effect of time. The cortisol level did not differ between times or groups (see Table S.3 in Supplementary Information for details), and correlated neither with subjective ratings nor evoked potential amplitudes.

### Correlations

Chlorpromazine equivalences, clinical symptoms or demographic variables did not correlate clearly with subjective and objective evaluations. There were some tendencies towards correlations between positive symptoms and physiological responses to electrical stimulations. Only one correlation was significant, however, between the Panss positive score and the amplitude of the P2 observed after electrical stimulations (electrode Cz) (N = 21, r = 0.45, p = 0.036). This correlation would not withstand correction for multiple measures. No correlation was significant between subjective evaluations of pain and emotion, nor between objective responses to electrical stimulations and negative pictures.

## Discussion

Overall, our results counter the belief that patients are generally unresponsive to unpleasant stimuli. There was no difference between groups in terms of their subjective pain ratings, and patients felt more aroused than controls after neutral and negative pictures. These results indicate that responses to unpleasant stimuli can be in the normal range or even higher than controls. Moreover, the amplitude of SEPs following electrical stimulations was significantly greater in patients than controls. Patients also displayed greater ERP amplitudes than controls in response to emotional pictures. Heightened sensitivity to emotional pictures and electrical stimulations cannot be explained away by a non-specific attention deficit or general deficit because both should have produced the opposite pattern of results. An effect of antipsychotic drugs also seems unlikely given the studies suggesting that pain perception is similar in treated and non-treated patients^14^,^16^,^26^, that emotional processing is normalized rather than worsened by antipsychotics^27^, and that antipsychotics are analgesic^28^. However, we cannot rule out the possibility that the response to stimulations was dampened on a subjective level by the intake of antipsychotics. Further studies should also look for a possible difference between antipsychotics. Finally, it is to be noted that our patient group was not preserved from impairments, insofar as the same sample of patients was very impaired at ordering information^29^.

There is an internal consistency in our results, in that all measures indicate either no difference between patients and controls, or a heightened response in patients. Furthermore, individual analyses suggest no patient showed a decreased response to electrical stimulations. When compared to the literature, these results may appear surprising at first sight, but a more in-depth results analysis may suggest the inconsistencies are more superficial. In fact, our results are consistent with the recent literature. For example, several studies have suggested patients are very sensitive to the emotion conveyed by visual information^30^,^31^. Controls, unlike patients, it is suggested, down-regulate their neural response to emotional images when informed or primed in advance about the valence of the next stimulus^23^,^32^. This might explain our findings. In our paradigm a majority of figures were negative (2/3) and thus predictable, which might have helped controls, but not patients, to down-regulate their emotional response.

Electrical stimulations were below pain threshold and results cannot be generalized and easily compared with previous studies on pain. It can be noticed, though, that the present study is not inconsistent with recent results if account is taken of the distinction made by Lévesque^17^ between acute (without sensitization) and chronic or repetitive pain (with sensitization). For example, the study of Girard *et al*.^33^ used pressure tests and the authors took care to avoid local sensitization by changing stimulation sites. They found that patients with schizophrenia were hypersensitive to pain. Increased activation of the primary somatosensory cortex and superior prefrontal cortex (but decreased activation in the posterior cingulate cortex and the brainstem) has been described in patients in response to painful thermal stimuli^34^,^35^. The greater amplitude found in frontal areas may correspond to the effects we measured 50 ms after stimulus onset. The amplitude reduction in the posterior cortex and brainstem does not match our own results, but this difference might be linked to differences in protocols. A 30s thermal pain administration may have induced sensitization^34^, whereas we avoided sensitization by using 30 s intervals between stimulations.

In our results, the early responses (50 ms after stimulation) are amplified in patients when compared to controls, suggesting that sensory processing already starts to differ between patients and controls at the earliest processing levels. Interestingly, Bak *et al*.^36^ showed abnormally high amplitudes of P50 in the context of sensory gating due to a lack of suppression of early signals. The paradigm in the present study is evidently different, not only in terms of the kind of stimulations, but also because the intervals between the electrical stimulations are much longer than in sensory gating paradigms (30 s vs. 500–1000 ms). Yet, our results are consistent with the idea that patients with schizophrenia have difficulty filtering incoming information^37^. This hypothesis may be further supported by the second SEP peak, the P1*. Its latency and spatial distribution are reminiscent of the well-known P3a which has previously been correlated with the novelty of a stimulus^38^. The presence of the P1* in patients and its absence in control subjects may indicate that patients process each stimulation as a new and unpredictable event, whereas controls would anticipate this information and thus not label it as a novelty, particularly as each electrical stimulation had been announced verbally. Finding a deficit in anticipation in patients would be consistent with a number of empirical results^39^,^40^,^41^,^42^. An increase in the amplitude of the P1* may be surprising in patients with schizophrenia, insofar as the P3a amplitude is usually described as decreased, especially during the oddball test^43^. Yet, this discrepancy with the literature is only superficial, and can be explained by the key differences between the oddball test and our own tasks. During the oddball test, the P3a is elicited by an effect of surprise due to an unexpected event occurring within a series of regular and predictable events. With our task, however, the target stimulation is predictable, and in fact no P3a was expected, and none was observed in controls. The peak observed in patients at 250 ms is thus an abnormal reaction to a predictable event, rather than a normal orientation of attention towards a deviant. Consistent with aberrant saliency^44^, it would reflect an abnormal attention orientation towards repetitive stimulations. This interpretation is all the more plausible given that we prepared the subjects, so as to avoid any nocebo effects^45^,^46^. On the first day, we reassured them by stressing that the stimulations were mild and by actually showing what they were like. Modulation of pain perception by top-down control appears to involve complex mechanisms including basic inhibition as well as high-level cognition^24^,^25^,^47^. Our method may thus have helped control subjects to gate sensory information, or to desensitize their response, and these control mechanisms may not have worked as well in patients with schizophrenia^48^.

Regardless of the mechanisms underlying these impairments, our study showed that stabilized patients with schizophrenia can react more intensely to acute electrical stimulations and negative pictures than healthy controls. Further studies are needed to check to what extent regulation mechanisms are related or not for emotion and electrical stimulations, and whether or not the lack of effect on subjective responses is due to a floor effect or a dissociation between physiological and subjective responses. It is important to note that these results cannot be generalized to include patients in acute phases who self mutilate, or chronic pain, which might involve other regulation mechanisms, like sensitization. Hence, our results do not signify generalized overreactivity. Our sample of patients was relatively small, limiting the conclusions that can be derived from the present study, although the individual analyses (see Supplementary Material) and effect sizes suggest the results are reliable (especially for the P1* and LPP). A larger sample of patients may be especially helpful for checking for correlations between clinical symptoms and increased physiological responses, as well as an in-depth analysis of the impact of antipsychotics.

However, our results do reinforce the idea that patients can be abnormally sensitive to unpleasant stimulation, which would become abnormally salient^44^,^49^. Interestingly, this was the case with mild stimuli. The electrical stimulations were below the pain threshold, and the pictures presented were only mildly negative. The intensity of the stimulations was not large enough to affect the stability of hormones mediating stress. Nonetheless the results suggest that aversive stimuli like low intensity electrical stimulations, or mildly negative pictures affect patients more than they can say. This might represent a vulnerability persisting throughout chronic states. From a clinical point of view, this means the lack of emotional responses in chronic patients should not prevent caregivers from trying to protect them from unpleasant events and stimuli. The possibility that patients may be more sensitive than they appear to be should be borne in mind. The present study suggests additional research is required to check to which amount our results generalize to pain above threshold, and whether care should be taken when treating patients’ pain, e.g. during anaesthesia.

## Material and Methods

### Participants

Each group consisted of 21 individuals. Controls were individually matched to patients in terms of gender, level of education, and age (all F’s < 1; Table 3).

All patients were in a stable state and fulfilled the criteria for the diagnosis of schizophrenia^50^ (see Table 3 for additional details, i.e. mean disease duration, mean age at onset, mean number of hospitalizations). Psychiatric diagnoses and the Positive and Negative Syndrome Scale (PANSS)^51^ scores were established by senior psychiatrists from the Psychiatry Departments of the Universities of Strasbourg and Besançon on the basis of semi-structured interviews and the MINI^52^. Exclusion criteria for patients and controls were: the intake of benzodiazepines and painkillers, a history of alcohol and drug dependency, neurological and medical pathologies (especially diabetes), a disabling sensory disorder, and general anesthesia in the 3 months prior to testing. An additional exclusion criterion for controls was psychotropic medication in the 3 weeks prior to testing.

A urine sample from each subject was analyzed to eliminate the presence in their system of opiates, benzodiazepines, and cannabis. All subjects had normal or corrected-to-normal visual acuity^53^.

Six patients were treated with anti-depressant drugs, but their results did not differ from those of the other 15 patients. The results displayed are averaged over the whole patient group. We recorded past painful events in their medical history, and patients with schizophrenia had undergone surgery slightly more often than controls (1.2 on average in patients vs 0.6 in controls, F[1,40] = 7.5, p < 0.01), and, as expected, reported more suicide attempts than controls (1 on average in patients vs. 0 in controls, F[1,40] = 6.8, p < 0.01). However, these parameters did not correlate with the results. For all other events, there was no significant difference between patients and controls (see Table 3).

The project was approved by the Ethics Committee (CPP EST IV in Strasbourg, France), and informed written consent was obtained from each subject. The study was carried out in accordance with the recommendations of the Declaration of Helsinki.

### Assessment of responses to electrical stimulations

In order to apply electrical stimulation, two electrodes were glued about 3 cm apart to the back of the subject’s hand before the experiment started. Each subject underwent two series of 20 successive stimulations. Each stimulation was announced 3 s in advance and followed by a 30 s interval (i.e. long enough to avoid sensitization phenomena). Participants rated the stimulation on two successive visual to analogue scales digitized from 0 to 100 (painfulness = no pain (0) to unbearable pain (100), unpleasantness = very unpleasant (0) to very pleasant (100)). For the sake of simplicity, the unpleasantness scale is inverted in the Results section.

Each electrical stimulation was a 50 ms sinusoidal signal with an optimal frequency of 5 Hz for activating the nociceptive C fibers^54^,^55^. The two stimulation series differed only in terms of intensity (1300 μA and 1800 μA) and were separated by an interval of about 40 minutes. All intensities were below the pain threshold.

### Tactile sensitivity assessment

Impaired tactile sensitivity might be a confounding factor for the perception of electrical stimulations. We used von Frey filaments to evaluate tactile sensitivity (see details under Supplementary Information).

### Pictures ratings

We tested subjects’ reactions to aversive stimuli with three series of 20 pictures from the International Affective Picture System (IAPS): negative with a high arousal level vs negative with a low arousal level vs neutral pictures^56^.

The pictures were displayed for 5 seconds in the center of a black screen, and subjects had to rate valence and arousal levels for each picture on a scale of 1 to 9 (see details under Supplementary Information).

### Procedure

Each subject participated in two sessions that took place on two consecutive days. On the first day we checked for visual acuity, color vision, tactile sensitivity, diabetes, and substance use, and tried to reassure the participants by letting them try 6 electrical stimulations, including 1800 μA.

**Figure 3:**
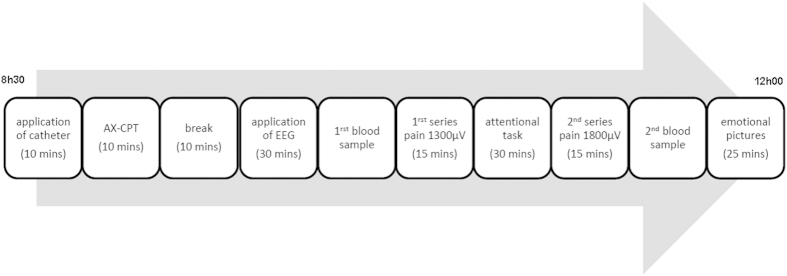
Experimental procedure performed on Day 2. For the sake of simplicity, we have not reported the results of the attentional tasks (AX-CPT), because they do not provide any relevant information. Early application of the catheter avoided any interference with blood sampling by the needle prick. The blood samples were taken twice, before and after the series of pain stimulations. The test involving emotional pictures was conducted at the end of the protocol, after the second blood sample was taken. About a quarter of an hour elapsed therefore between the last series of electrical stimulations and the presentation of the pictures.

The main part of the experiment took place the following morning. Due to the circadian variations of the hormones measured (ACTH and Cortisol), all subjects underwent the different tests and provided blood samples at the same time of the day (Fig. 3) (see Supplementary Information for details).

### Electrophysiological recordings

We recorded brain activity with an EEG throughout the emotion and pain assessments (see Supplementary Information for technical details)^57^.

In the pain condition with minimal intensity, EEG recordings included too many artefacts in 6 subjects (3 patients/3controls), who were thus excluded from the analysis of 1300 μA stimulations. For the same reason, 4 subjects (2 patients/2 controls) were excluded from the EEG analysis of emotional pictures. No data were excluded in the critical pain condition, with 1800 μA stimulations.

### Analysis of pain-related SEPs

We selected single EEG epochs from 200 ms before (baseline correction) to 800 ms after pain stimulation in order to measure and compare peak amplitudes of early and late event related potentials.

Our analysis was focused on spatio-temporal regions of interest (ROI) typically related to pain: N1 [80–200 ms], P1 [150–210 ms] and N2/P2 [200–500 ms] at the site Cz^58^.

We analyzed two additional SEP components observed in the group of patients: a very early signal, i.e. the P50 [10–60 ms], and a later positive component around 210–250 ms after the stimulations. For each participant we assessed individual peaks (amplitude and latency) at the chosen spatial and temporal regions of interest.

### Visual ERPs

We defined EEG epochs from 200 ms before (baseline correction) to 2000 ms after picture presentation onset to measure early and late visual ERPs in response to the three different picture types.

We analyzed spatio-temporal regions of interest typically related to emotion processing^59^,^60^: an early negative component between 100 and 200 ms, a complex consisting of a positive peak and a negative peak between 200 and 400 ms, and a late positive potential (LPP). Since this late potential was long in duration, from 600 to 1600 ms after picture onset, it was analyzed in successive 200 ms bins^59^.

## Additional Information

**How to cite this article**: Duval, C. Z. *et al*. Neurophysiological responses to unpleasant stimuli (acute electrical stimulations and emotional pictures) are increased in patients with schizophrenia. *Sci. Rep.* **6**, 22542; doi: 10.1038/srep22542 (2016).

## Supplementary Material

Supplementary Information

## Figures and Tables

**Table 1:** Early and late pain-related SEP measurements at the sites Cz and Fz, with mean amplitudes of the evoked potentials (with standard errors).

	**mean (SEM) patients**	mean (SEM) controls	F	Df	P	Cohen’s d
Cz						
P50						
Main effect: Group						
1300μA	3.98 (1.3)	1.3 (0.6)	3.62	1,34	0.07	–
1800μA	3.65 (1.1)	1.25 (0.3)	4.17	1,40	< 0.05	0.62
N1						
Main effect: Group						
1300μA	−4.9 (1.7)	−3.69 (1)	0.38	1,34	0.54	–
1800μA	−4.22 (1.7)	−1.56 (0.6)	3.15	1,40	0.08	–
P1						
Main effect: Group						
1300μA	6.15 (1.1)	3.28 (1.3)	2.93	1,34	0.1	–
1800μA	6.28 (1.2)	4.74 (0.9)	1.13	1,40	0.29	–
P1*						
Main effect: Group						
1300μA	3.87 (1.6)	−0.88 (2.1)	3.25	1,34	0.08	–
1800μA	4.97 (2)	−0.27 (1.2)	6.47	1,40	< 0.05	0.7
N2						
Main effect: Group						
1300μA	−6.2 (1.4)	−6.59 (2.1)	0.02	1,34	0.88	–
1800μA	−6.33 (1.3)	−6.04 (1.2)	0.02	1,40	0.88	–
P2						
Main effect: Group						
1300μA	16.27 (1.2)	15.52 (1.5)	0.14	1,34	0.71	–
1800μA	11.71 (1.4)	11.68 (1.1)	0.0003	1,40	0.99	–
Fz						
P50						
Main effect: Group						
1300μA	4.6 (1.4)	1.81 (0.8)	2.94	1,34	0.1	–
1800μA	4.16 (1.1)	1.72 (0.4)	4.4	1,40	< 0.05	0.67
P1*						
Main effect: Group						
1300μA	5.34 (1.6)	−1.1 (1.8)	7.22	1,34	< 0.05	0.9
1800μA	6.51 (2.2)	−1.2 (1.2)	9.18	1,40	< 0.005	0.94

The statistics of group effects are detailed for each evoked potential following the 1300 and 1800 μA stimulations at Cz, and the P50 and P1* at Fz. The p significance levels were set at p < 0.05, and the Cohen’s d value shows the effect size.

**Table 2:** Mean ERP peak amplitude (with standard errors) in the two groups after picture presentation.

	mean (SEM) patients	mean (SEM) controls	F	Df	P	Cohen’s d
Fz						
P1 (0.2–0.4 s)						
Main effect: Group (μV)	0.54 (1.0)	0.95 (1.0)	0.14	1,36	0.71	–
N2 (0.2–0.4 s)						
Main effect: Group (μV)	−8.1 (1.0)	−8.13 (1.2)	.0005	1,36	0.98	–
LPP (1.0–1.2 s)						
Main effect: Group (μV)	5.55 (1.0)	3.5 (1.1)	4.34	1,36	< 0.05	0.57
LPP (1.2–1.4 s)						
Main effect: Group (μV)	5.36 (0.8)	2.83 (1.0)	7.48	1,36	< 0.05	0.62
LPP (1.4–1.6 s)						
Main effect: Group (μV)	4.67 (0.8)	1.91 (1.1)	9.26	1,36	< 0.005	0.64
Cz						
P1 (0.2–0.4 s)						
Main effect: Group (μV)	2.04 (0.7)	2.03 (0.8)	0.00002	1,36	0.99	–
N2 (0.2–0.4 s)						
Main effect: Group (μV)	−6.3 (0.9)	−6.07 (1.1)	0.03	1,36	0.87	–
LPP (1.0–1.2 s)						
Main effect: Group (μV)	3.5 (0.8)	2.08 (1.0)	3.66	1,36	0.06	–
LPP (1.2–1.4 s)						
Main effect: Group (μV)	3.36 (0.8)	1.46 (0.9)	6.15	1,36	< 0.05	0.53
LPP (1.4–1.6 s)						
Main effect: Group (μV)	3.48 (0.5)	1.05 (0.6)	9.26	1,36	< 0.005	1.04

The ERP results are averaged and combined over all three picture types. The statistics of group effects are detailed for evoked potential at Cz, and Fz. The p significance levels were set at p < 0.05, and the Cohen’s d value shows the effect size.

**Table 3:** Demographic and clinical data about the participants, including the average number of painful events in their medical history.

	Patients	Controls
Gender (M/F)	16/5	16/5
Age (mean ± SD)	37.7 ± 9.2	37.4 ± 10.7
Years of education (mean ± SD)	13.3 ± 2.3	13.1 ± 2.3
Medication (typical/atypical/no medication)	5/14/2	─
Dose of chlorpromazine equivalents	244 mg/day	─
Anti-Parkinsonian treatment (tropatepine)	4	─
Number of hospitalizations in psychiatry	3.3 ± 3	─
Mean age at disease onset	25.2 ± 5	─
Mean disease duration	12.2 ± 7	─
Outpatients/Inpatients	20/1	─
PANSS positive symptoms (mean ± SD)	17.5 ± 5.9	─
PANSS negative symptoms (mean ± SD)	21.9 ± 8.2	─
PANSS general symptoms (mean ± SD)	38.2 ± 10.4	─
PANSS total (mean ± SD)	77.6 ± 10.1	─
Mean number of medical painful events (mean ± SD)	0.8 ± 0.8	0.8 ± 1
Mean number of surgeries (mean ± SD)	1.2 ± 0.9	0.6 ± 0.6
Mean number of accidents (mean ± SD)	1 ± 0.9	1.4 ± 1.2
Mean number of suicide attempts (mean ± SD)	0.4 ± 0.7	0
