# Supplementary material for: Neurophysiological responses to unpleasant stimuli (acute electrical stimulations and emotional pictures) are increased in patients with schizophrenia
Source: Sci Rep. 2016 Mar 3;6:22542. doi: 10.1038/srep22542 (PMC4776095; doi:10.1038/srep22542)
Supplement: Supplementary Information [file srep22542-s1.pdf]

Neurophysiological responses to unpleasant stimuli (acute electrical stimulations and emotional pictures) are increased in patients with schizophrenia

Dr. Céline Z. Duval[1,2,3], Dr. Yannick Goumon[4], Dr. Véronique Kemmel[5,6], Prof. Jürgen Kornmeier[7,8], Prof. André Dufour[9], Dr. Olivier Andlauer[10], Prof. Pierre Vidailhet[1], Prof. Pierrick Poisbeau[4], Dr. Eric Salvat[4,11], Prof. André Muller[4,11], Prof. Ayikoé G. Mensah-Nyagan[5], Dr. Catherine Schmidt-Mutter[12], Dr. Anne Giersch[1]

1. INSERM U-1114, Fédération de Médecine Translationnelle de Strasbourg (FMTS), Département de Psychiatrie, Hôpitaux Universitaires de Strasbourg; 1, place de l'Hôpital, 67000 Strasbourg, France

2. Fondation FondaMental, Créteil, France

3. Fondation APICIL, 21, place Bellecour, 69002 Lyon

4. Institut des Neurosciences Cellulaires et Intégratives, CNRS UPR 3212, 5, rue Blaise Pascal, 67084 Strasbourg, France

5. INSERM U-1119 Biopathologie de la Myéline, Neuroprotection et Stratégies Thérapeutiques, Université de Strasbourg, Bâtiment 3 de la Faculté de Médecine, 11 rue Humann, 67 000 Strasbourg, France.

6. Laboratoire de Biochimie et Biologie Moléculaire, Hôpitaux Universitaire de Strasbourg, 67098 Strasbourg, France

7. Institute for Frontier Areas of Psychology and Mental Health, 79098 Freiburg, Germany

8. University Eye-Hospital, Killianstraße 5, 79106 Freiburg, Germany

9. Laboratoire de Neurosciences Cognitives et Adaptatives, UMR 7364, Université de Strasbourg, CNRS, Strasbourg, France

10. EA 481 Laboratoire de Neurosciences, Université de Franche-Comte, 1 place du maréchal Leclerc, 25030 Besançon Cedex, France

11. Centre 'Evaluation et de Traitement de la Douleur (CETD) du CHRU, Hôpital de Hautepierre, 1 av Moliere, 67098 Strasbourg, France

12. INSERM Centre d'investigation clinique-1434, CHRU, 1, place de l'Hôpital, 67000 Strasbourg, France

Supplementary information 1

Material and Methods

Von Frey filaments

This test consists of varying diameters and toughness (in gram) of nylon filaments, which are pressed against the skin until the filament is curved. We chose 10 different diameters which were each tested 20 times on the back of the subject's hand, in random order. The intensity of the stimulation increases with the diameter of the filaments, such that it is possible to quantify subjects' touch sensitivity. It should be emphasized that these filaments do not induce pain, and only allowed us to check for tactile sensitivity (unlike the filaments used in animal research). Subjects were asked to close their eyes and to signal verbally when they felt they had been touched.

Emotional pictures ratings

The experiment was run on a Pentium 4 PC. The stimuli were displayed on an Iiyama monitor (21-inch, 85 Hz refresh rate). E-prime 2 was used to automatize the whole procedure, and the order of the pictures was semi-randomized. Each picture remained on the screen for 5 seconds. After each picture, subjects rated its valence and arousal strength according to the SAM visual analogue scales also presented on the computer screen. The SAM consists of a row of graphic figures that range from smiling and happy to frowning and unhappy, with 9 levels ranging from 1 to 9 to represent the hedonic valence dimension. For the arousal dimension, SAM figures range from relaxed and sleepy to excited and wide-eyed, again with 9 levels from 1 to 9[1]. As soon as the rating was complete the next picture was displayed.

The mean emotional valence and arousal levels of the pictures displayed were the following:

[1] negative valence (3.6 SD 1.9), high arousal level (6.2 SD 2.1),

[2] negative valence (3.6 SD 1.7), lower arousal level (4.4 SD 2.2)

[3] neutral pictures (valence = 5.0 SD 1.2; arousal = 2.9 SD 1.9).

EEG recording

We used a Biosemi® active-two device to record EEG, with 64+8 multi-channel AgCl active electrodes. 64 electrodes were mounted on an elastic cap and distributed according to the international 10/20 system

The EEG signal was sampled at the rate of 512 Hz (0.01–100 Hz bandpass filter, 12 dB/octave), and the reference electrodes were located at both earlobes (averaged off-line). Eye movement artifacts were monitored with additional electrodes, measuring vertical and horizontal electrooculographic bipolar potentials, and EEG recordings were corrected off-line with an Independent Component Analysis procedure[2,3]. Brainvision Analyzer software (Brainproducts, Munich, Germany) was used to process the results off-line.

SEP peaks were measured relative to a baseline, defined as the average amplitude of data points 200 ms before onset of pain stimulation.

Latency shift analysis

In a first step, we determined the latency of our evoked potentials automatically based on the Brain Analyzer, which did not show any group difference. However, it seemed important to check for a possible global time lag in patients, since this would have led to contrast an early heightened response with a late delayed response. In order to test for ERP latency differences

between patients and controls, we thus used an additional approach, based on cross-correlations of grand mean data, combined with permutation tests:

(1) Cross-correlation

(1.1) As spatial ROI we chose again the Cz and Fz electrodes. For the temporal ROI we calculated two variants:

- A temporal window between t0 = 0 (stimulus onset) and t1 = 300 ms, encompassing the early P50 and the subsequent N100, P200 and N300 but excluding the late slow positivity.

- A temporal window between t0 = 0 (stimulus onset) and t1 = 600 ms, encompassing both the early ERP components and also the slow positivity.

The P3b is a late and large positive excursion that can easily dominate any correlation calculation, in particular if this component covers a considerable part of the temporal ROI. Based on these considerations we decided to analyze the latency shift with and without the P3b-like late positivity.

(1.2) Grand mean data of the control participants within the above-described spatiotemporal ROI were used as template data. Corresponding patient grand mean data were cross-correlated with these template data within a cross-correlation range of ± 120 ms with respect to the template trace. The latency delay providing the maximal correlation value was determined as the estimate of the potential latency delay of patient ERP traces compared to control traces.

(2) Statistical analysis

(2.1) For one specific electrode position we shuffled the ERP traces from all participants, resorted them randomly into two groups and calculated grand mean traces for each of the two arbitrary groups.

(2.2) We calculated cross-correlations of the two randomly generated grand mean traces, as described above. The maximal correlation value was stored.

(2.3) We performed this random grouping and cross-correlation calculation 100 times, then calculated a distribution of the resulting maximal correlation coefficients. A p-value was calculated as the subspace under this distribution as determined by the position of the original correlation coefficient (based on the original patient-control grouping) within this distribution.

Blood sample

Blood was sampled in lithium heparin tubes (LH-PSTII tubes; Becton Dickinson, Le Pont de Claix, France) before and after the electric stimulations. When subjects arrived for the experiment a catheter was inserted in the crook of one of their arms to prevent any interference from the pain induced by the needle prick during blood sampling. The first blood sample was taken 50 min after the catheter had been inserted.

ACTH and Cortisol concentrations were determined by means of electrochemiluminescence immunoassay on Cobas E 601 (Roche Diagnostics, Indianapolis, Indiana, USA) according to the manufacturer’s instructions. The intra and inter-day accuracy of the methods used was between 5 % and 10% for the routine quality control.

Supplementary information 2

Results

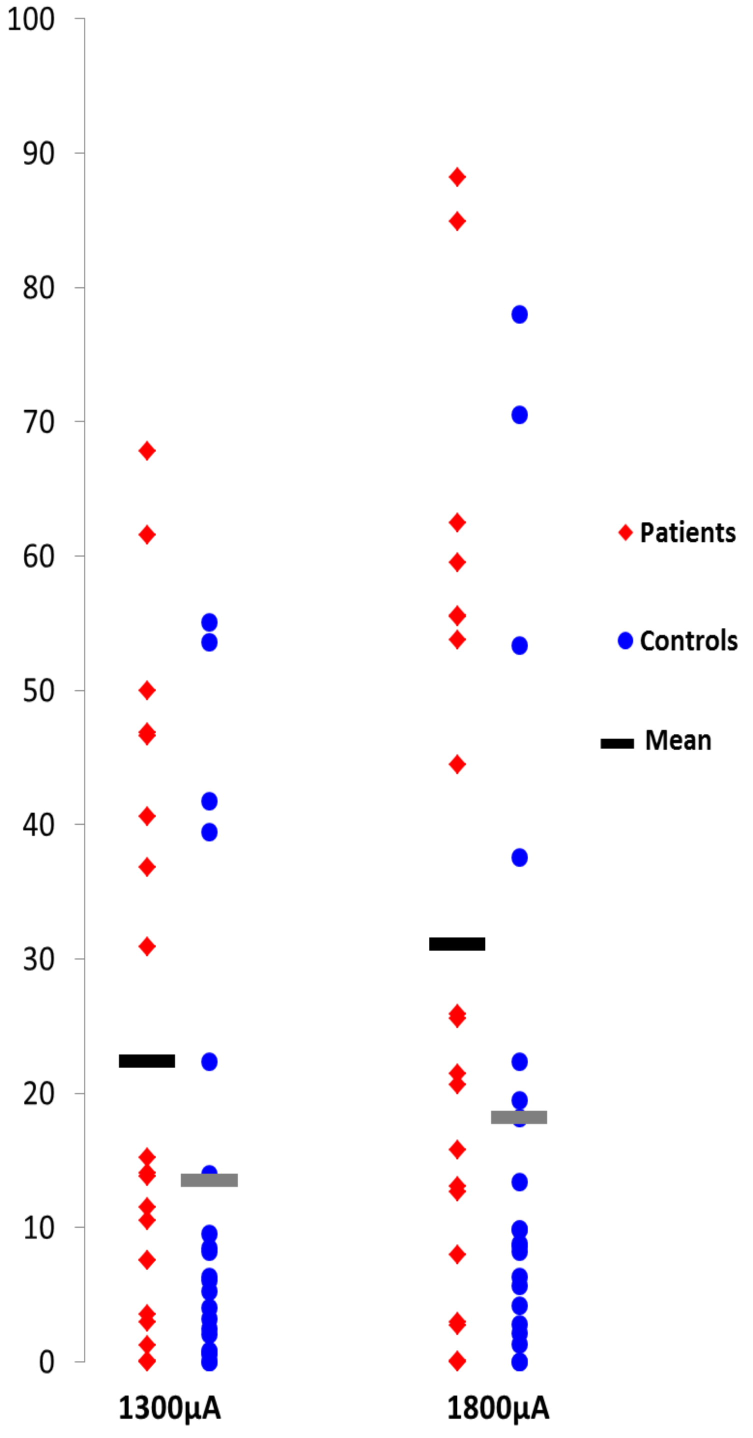

S1. Individual subjective pain evaluations data in both pain conditions. Mean data for each group is shown as a black bar.

Individual analyses

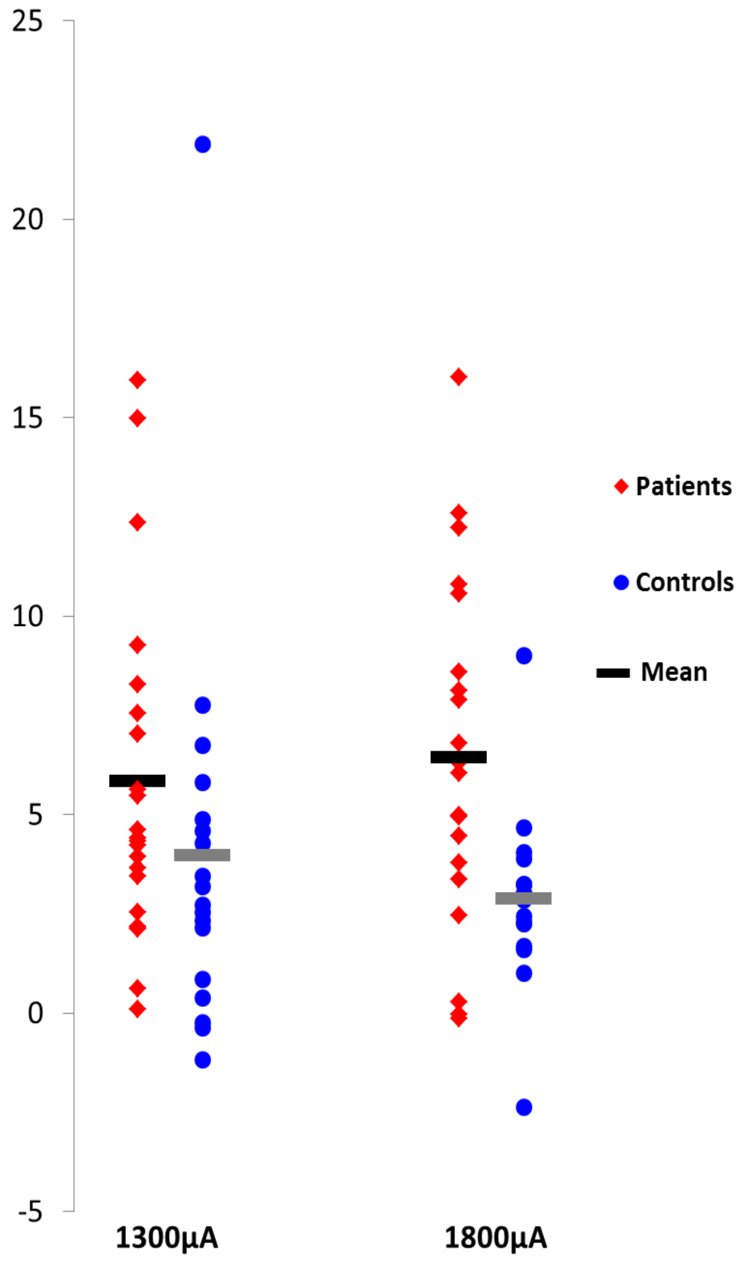

S2. Individual P50 amplitude data in both pain conditions. Mean data for each group is shown as a black bar. These results show that no patient displays a physiological response below the range of responses in healthy volunteers. Like for subjective responses, these results show that in this precise experiment, there is no sub-group of patients showing a reduced sensitivity to electrical stimulations relative to controls.

Latency shift results:

Correlation shift window: ± 120 ms

Temporal ROI for template trace: 0 – 600 ms (including P3b):

Cz: latency shift for maximal correlation: 32 ms; $r = 0.95$; $p = 0.41$

Fz: latency shift for maximal correlation: 52 ms; $r = 0.85$; $p = 0.64$

Temporal ROI for template trace: 0 – 300 ms (excluding P3b):

Cz: latency shift for maximal correlation: 24 ms; $r = 0.67$; $p = 0.76$

Fz: latency shift for maximal correlation: 56 ms; $r = 0.51$; $p = 0.97$

Cross-correlation calculations provided latency delays of patient ERP data compared to healthy control ERP data between around 25 and 55 ms (between 24 and 36 ms at the Cz electrode and between 52 and 56 ms at the Fz electrode). Neither of these latency delays was indicated as significant.

Tactile sensitivity

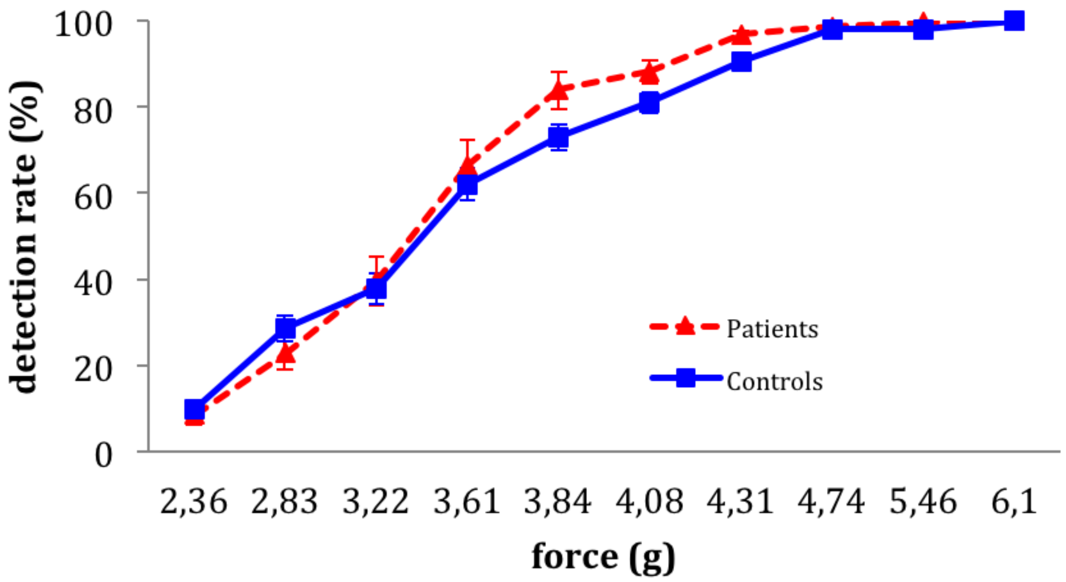

S3. Detection rate of tactile sensation as a function of the pressure (in gram) of the von Frey filaments (patients vs controls).

Blood sample results

	mean (σ) patients	mean (σ) controls	p-values (patients vs. controls)
Cortisol (nmol/L)			
Before stimulation	424.7 (189.8)	361.9 (140.2)	0.17
After stimulation	413.0 (151.3)	368.2 (103.7)	0.33
p- values (before vs. after)	0.80	0.89	
ACTH (pmol/L)			
Before stimulation	6.33 (3.8)	4.77 (1.9)	0.22
After stimulation	6.85 (6.69)	4.76 (2.0)	0.10
p- values (before vs. after)	0.68	0.99	

S4. Comparison of blood sample results between patients and controls based on an ANOVA and a subsequent Fisher’s LSD post hoc test.

References

1. Bradley, M.M., & Lang, P.J. Measuring emotion: The self-assessment manikin and the semantic differential. *J. Behav. Ther. Exp. Psychiatry*. **25**, 49–59 (1994).

2. Hoffmann S, Falkenstein M. The correction of eye blink artefacts in the EEG: a comparison of two prominent methods. *PloS One*. **3**, (2008).

3. Jung TP, Makeig S, Westerfield M, Townsend J, Courchesne E, Sejnowski TJ. Removal of eye activity artifacts from visual event-related potentials in normal and clinical subjects. *Clin. Neurophysiol*. **111**,1745–58 (2000).
